# Novel plant cell suspension platforms for saffron apocarotenoid production and its impact on carotenoid and volatile profiles

**DOI:** 10.1111/pbi.70153

**Published:** 2025-06-19

**Authors:** Maria Lobato‐Gómez, Markus Laurel, Marta Vázquez‐Vilar, José L. Rambla, Diego Orzáez, Heiko Rischer, Antonio Granell

**Affiliations:** ^1^ Instituto de Biologíıa Molecular y Celular de Plantas CSIC‐Universidad Politécnica de València Valencia Spain; ^2^ VTT Technical Research Centre of Finland Ltd. Espoo Finland; ^3^ Departamento de Biología, Bioquímica y Ciencias Naturales Universitat Jaume I Castellón de la Plana Spain

**Keywords:** plant cell suspension, crocins, picrocrocin, safranal, carotenoid cleavage deoxygenase, bioreactor

## Abstract

Saffron apocarotenoids, including crocins, picrocrocin and safranal, are valuable metabolites with pharmaceutical and cosmetic potential. However, their natural plant sources are difficult to cultivate, which limits large‐scale production. The identification of carotenoid cleavage dioxygenases (CCDs), which catalyse the first and most critical step in their biosynthesis, has enabled the production of these apocarotenoids in heterologous plant systems. In this study, we aimed to generate plant cell suspensions expressing *Crocus sativus* CCD2 and *Gardenia jasminoides CCD4a*, along with a bacterial phytoene synthase to enhance carotenoid biosynthesis and *CsUGT93P1*, which improves crocin stability. Transgenic cell suspensions were established from *Nicotiana benthamiana* plants and *Nicotiana tabacum* cv. BY‐2 cells. In BY‐2 cells expressing *GjCCD4a*, crocin accumulation reached 770 μg/g DW, which further increased upon methyl jasmonate elicitation. Remarkably, the BY‐2 transgenic cells exhibited an 18,000‐fold increase in β‐cyclocitral content compared to wild‐type cells. The best‐performing *N. benthamiana* and BY‐2 lines were successfully cultivated in wave bioreactors, demonstrating their potential for saffron apocarotenoid production. In the BY‐2 bioreactor, apart from saffron apocarotenoids, phytoene and notably high amounts of lycopene were produced, adding value to the platform and indicating a remodelling of the carotenoid pathway. This study establishes the viability and lays the foundation for the scalable production of saffron apocarotenoids and carotenoids in plant cell suspensions.

## Introduction

Saffron spice, obtained from the stigmata of *Crocus sativus*, is widely used for culinary purposes but also possesses several pharmacological properties, including antioxidant, anticancer and neuroprotective effects, attributed to the accumulation of its unique apocarotenoids: crocins, picrocrocin and safranal (Rameshrad *et al*., 2018; Ríos *et al*., 1996). However, the production of saffron is severely limited by the high manual labour required, resulting in elevated prices (Winterhalter and Straubinger, 2000).

**Figure 1:**
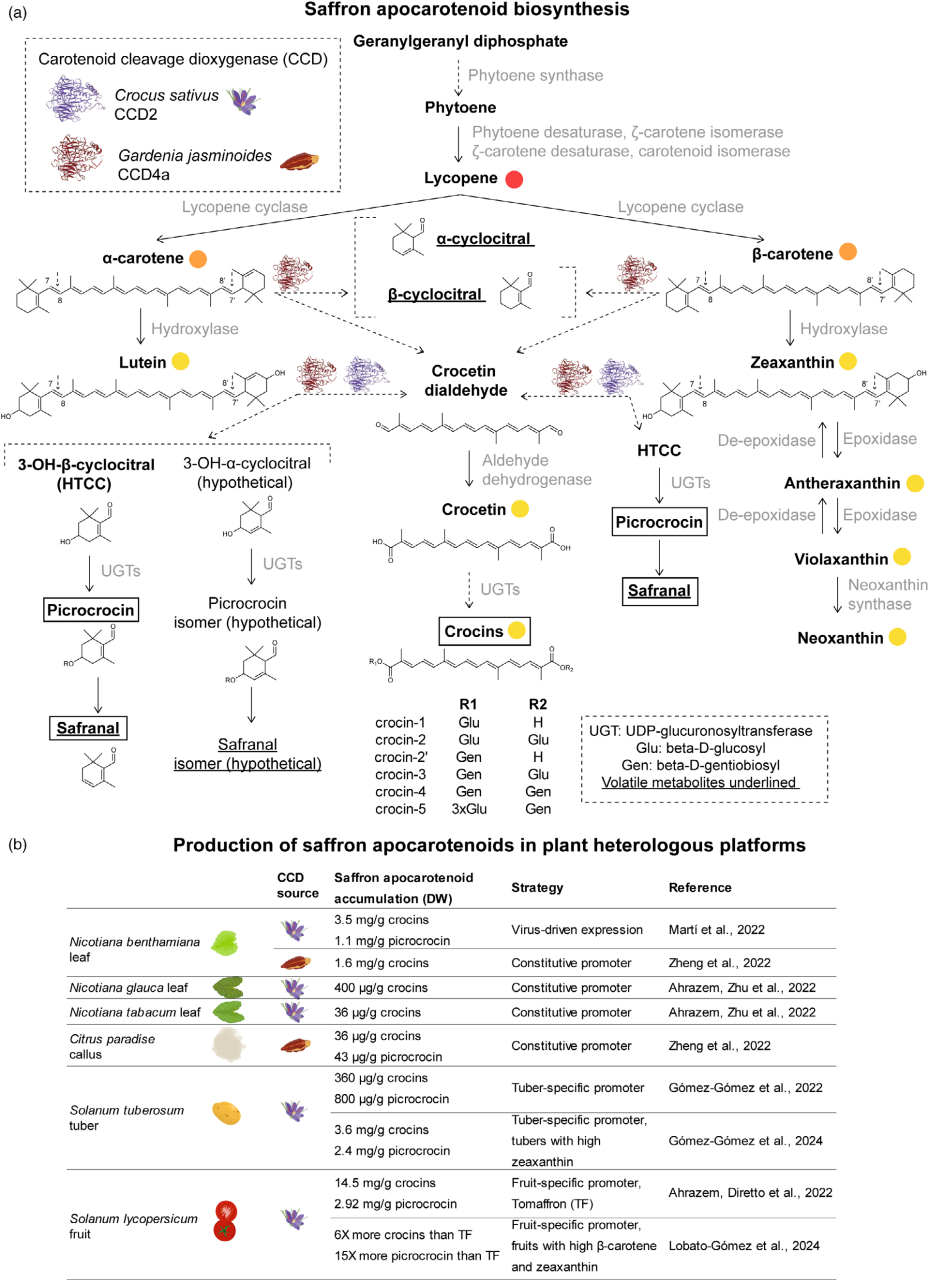
(a) Carotenoid and saffron apocarotenoid biosynthetic pathways. The enzymatic steps engineered in this work to produce crocins, picrocrocin and safranal are indicated with dashed arrows. Adapted from (Lobato‐Gómez *et al*., 2024; Zheng *et al*., 2022). (b) Summary of different strategies to produce saffron apocarotenoids in plant heterologous platforms.

Saffron apocarotenoids are not exclusively produced in *C. sativus* but also accumulate in *Buddleja davidii* flowers (Liao *et al*., 1999) and *Gardenia jasminoides* fruits (Pfister *et al*., 1996), which are also difficult to access. In *C. sativus*, the key step in saffron apocarotenoid biosynthesis is the enzymatic cleavage of zeaxanthin by carotenoid cleavage dioxygenase 2 (CCD2) (Frusciante *et al*., 2014) (Figure 1a). Identifying this rate‐limiting enzyme in the pathway has enabled its expression in heterologous platforms, overcoming the limitations of obtaining saffron apocarotenoids from their natural source.

The *C. sativus* CCD2 has been introduced into several plant heterologous systems (Figure 1b), resulting in saffron apocarotenoids accumulating in *Nicotiana* leaves (Ahrazem *et al*., 2022b; Martí *et al*., 2020), potato tubers (Gómez Gómez et al., 2022, 2024) and tomato fruit (Ahrazem *et al*., 2022a; Lobato‐Gómez *et al*., 2024). The CCD4a from *G. jasminoides* can also utilize β‐carotene as a substrate to produce saffron apocarotenoids (Figure 1a). This enzyme outperformed *CsCCD2* in *N. benthamiana* transient expression assays and citrus calli (Zheng *et al*., 2022).

Carotenoids accumulate naturally in tomato fruits and the tubers of some potato varieties. Thus, selecting cultivars or mutant plants with high levels of specific *Cs*CCD2 substrates has led to enhanced accumulation of saffron apocarotenoids (Gómez‐Gómez *et al*., 2024; Lobato‐Gómez *et al*., 2024; Morote *et al*., 2023). A well‐known example of engineering the carotenoid pathway is the development of Golden Rice, achieved by introducing exogenous phytoene synthase (*PSY; CrtB* when bacterial) and phytoene desaturase (*PDS; CrtI* when bacterial) genes (Paine *et al*., 2005; Ye *et al*., 2000). Similarly, in platforms with low carotenoid levels, boosting the pathway through the expression of exogenous phytoene synthase and/or β‐carotene hydroxylase (*BCH*; *CrtZ* when bacterial) genes has been combined with *CCD* expression to enable the production of saffron apocarotenoids (Ahrazem *et al*., 2022b; Martí *et al*., 2020; Zheng *et al*., 2022).

An alternative to producing saffron apocarotenoids in whole‐plant systems is using undifferentiated plant cells. Specialized metabolites can be efficiently produced in these systems under rapid growth conditions, a controlled environment and easy scaling‐up processes in a high level of containment that reduces the environmental concerns associated with transgenic plants (Fischer *et al*., 1999; Huang and McDonald, 2009; Rischer *et al*., 2020; Santos *et al*., 2016). Saffron apocarotenoids have been produced in transgenic rice and citrus calli but are limited to the objective of elucidating their biosynthetic pathway and metabolic implications (Ahrazem *et al*., 2016; Zheng *et al*., 2022).

An approach to obtaining cell suspensions producing saffron apocarotenoids could be the generation of undifferentiated cells derived from CCD‐engineered transgenic plant species with a good growth response *in vitro*, as has been previously done to obtain cell suspensions from *Nicotiana benthamiana* plants expressing antibodies against snakebite envenomation (Gomes *et al*., 2019) or cell cultures from *Nicotiana tabacum* transgenic plants accumulating geraniol (Vasilev *et al*., 2014).

Alternatively, cell cultures producing saffron apocarotenoids could be directly obtained by the stable transformation of well‐established cell suspensions, such as *N. tabacum* cv. Bright Yellow‐2 (BY‐2). BY‐2 cells exhibit rapid growth and have been successfully used to scale up the production of exotic proteins and metabolites (Huang and McDonald, 2009; Nagata *et al*., 2004).

In this work, we succeeded in producing saffron apocarotenoids in heterologous plant cell suspensions by transforming *N. benthamiana* plants and *N. tabacum* BY‐2 cells with *Cs*CCD2 and *Gj*CCD4a and evaluated the differences in their performance. Introducing these exotic CCDs led to the remodelling of the cell's carotenoid and volatile profiles, resulting in high β‐cyclocitral, phytoene and lycopene production. As a first step toward upscaling the production of saffron apocarotenoids, the best‐performing line from each platform was cultivated in a wave bioreactor. Cultivating plant cells producing saffron apocarotenoids and carotenoids provides an aseptic and contained alternative to whole‐plant systems.

## Results

### Generation of transgenic *Nicotiana benthamiana* plants

*N. benthamiana* leaf explants were transformed with the CCD2 and CCD4 cassettes containing the *C. sativus UGT91P3 and P. ananatis CrtB* genes in combination with either *CCD2* from *C. sativus* or CCD4a from *G. jasminoides*, respectively (Figure S1a). Several kanamycin‐resistant plants were recovered, and 11 plants from each transformation experiment were genotyped. Interestingly, many contained only the *CCD* transgene and not the other two transgenes. The kanamycin resistance gene (*nptII*) is located next to the left border and is the last element to be inserted into the plant DNA, followed by *CsCCD2* or *GjCCD4a*. The genotyping results suggest that deletions of the transgenes other than *nptII* could have occurred after T‐DNA integration into the plant genome (Figure S1a,b,d).

The genotyped transgenic lines were also screened for total crocin accumulation, but only three lines (CCD2 #3, CCD2 #10 and CCD4a #7) showed detectable crocin levels. The first two lines lacked both *PaCrtB* and *CsUGT91P3*, and accumulated very low levels of crocins in the T2 generation, where *CsCCD2* expression decreased significantly compared to the T0. On the contrary, the CCD4a #7 line contained all three transgenes, and the T2 still accumulated crocins (1.05 ± 44.19 mg/g DW), 3‐OH‐β‐cyclocitral or 4‐hydroxy‐2,6,6‐trimethyl‐1‐cyclohexene‐1‐carboxaldehyde (HTCC) (358.85 ± 41.21 μg/g DW) and picrocrocin (52.41 ± 4.34 μg/g DW). Despite this, gene expression analysis revealed significantly lower expression of both *GjCCD4a* and *PaCrtB* in the T2 generation compared to T0. Nevertheless, the expression levels of *GjCCD4a* remained high enough in the T2 plants to produce significant amounts of crocins. The decrease in *GjCCD4a* expression from T0 to T2 suggests transcriptional silencing of this gene in later generations (Figure S1e).

Taken together, the stable transformation of *N. benthamiana* using a strong constitutive promoter, such as 35S, does not seem feasible for crocin production in leaves. We assume this could be due to gene deletion upon T‐DNA integration into the plant genome and gene silencing caused by decreased transgene expression.

### Generation of cell suspensions from transgenic *N. benthamiana* plants accumulating crocins

**Figure 2:**
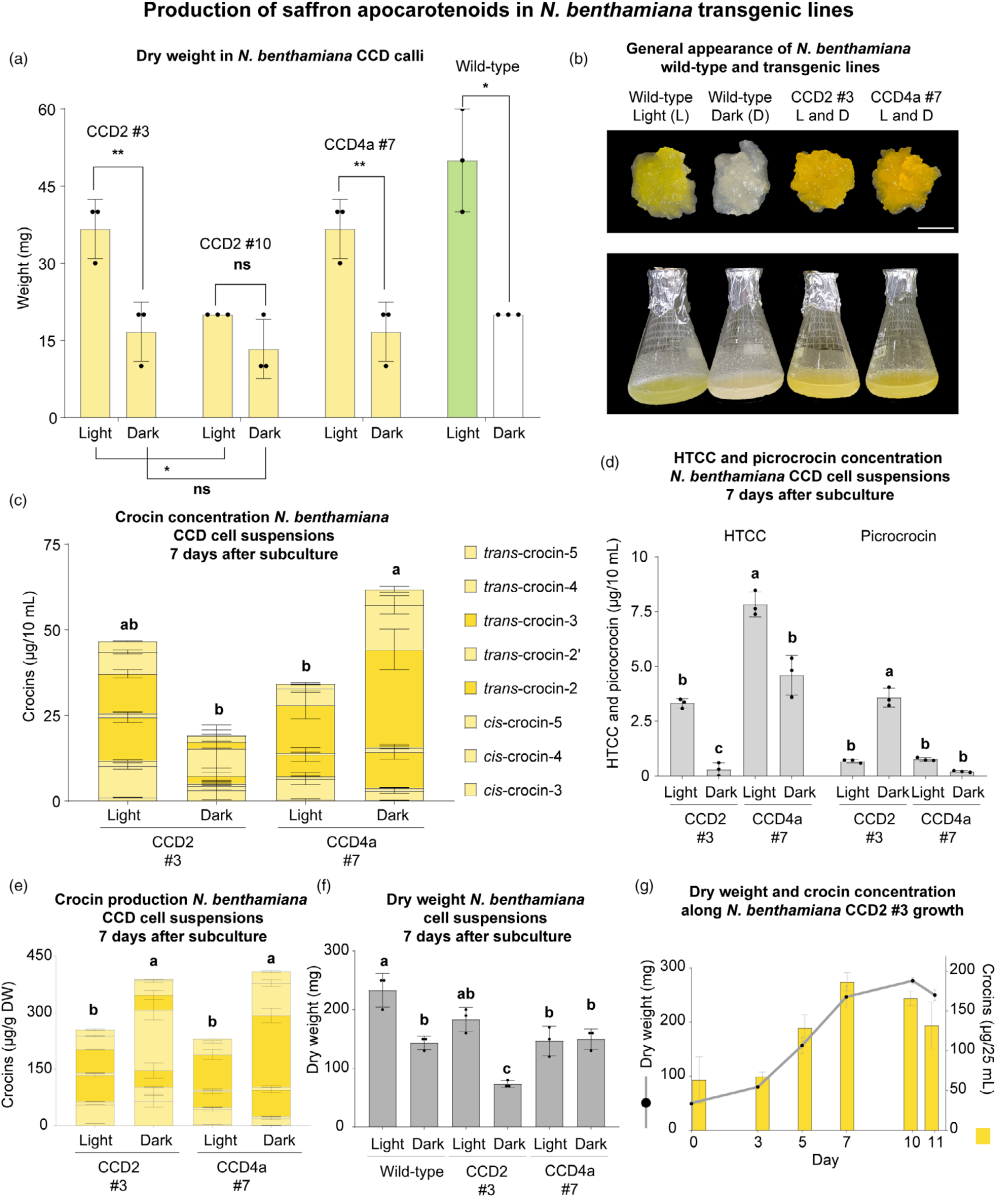
Characterization of *Nicotiana benthamiana* CCD cell lines. (a) Callus dry weight. (b) Phenotype of calli and cell suspensions. (c) Crocin, (d) HTCC and picrocrocin concentration. (e) Crocin production. Crocins, picrocrocin and HTCC were quantified from 7‐day‐old cell suspensions by LC–MS. None of these metabolites were detected in the wild‐type cell suspensions. *Cis*‐crocin‐1, *trans*‐crocin‐1 and crocetin were not detected. (f) Cell suspensions dry weight in 7‐day‐old cell suspensions. (g) Crocin yield and dry weight in the *N. benthamiana* CCD2 #3 growth curve under light conditions. Further details of the growth curve can be found in Figure S2. Bars represent the mean ± standard deviation (SD), with individual points indicating biological replicates. Statistical differences between two conditions were determined using a Student's *t*‐test (non‐significant (ns), *P* ≤ 0.05 (*), *P* ≤ 0.01 (**) and *P* ≤ 0.001 (***)). For comparisons involving three or more samples, a one‐way ANOVA was performed, followed by Tukey's post hoc test. Different letters above the bars indicate statistically significant differences (*P* < 0.05) among groups. CCD: carotenoid cleavage dioxygenase. HTCC: 3‐OH‐β‐cyclocitral.

Leaf explants from CCD2 #3, CCD2 #10, CCD4a #7 and wild‐type *N. benthamiana* T0 plants were surface‐sterilized and placed on callus induction medium to obtain friable calli that would allow the initiation of a cell suspension. The explants were distributed under light or dark conditions, and the growth in the two conditions was assessed by weighing the calli after two weeks. Overall, the calli grew faster in light conditions for all genotypes, and the calli from CCD2 #3 grew the fastest among the CCD2 transgenic lines (Figure 2a).

Cell suspensions from the wild‐type, CCD2 #3 and CCD4a #7 were established under light and dark conditions. Wild‐type calli and cell suspensions appeared green under light conditions and white in the darkness, respectively, whereas CCD2 #3 and CCD4a #7 exhibited yellow colour in both conditions (Figure 2b). Seven days after the subculture, CCD2 #3 produced higher concentrations of crocins under light than dark conditions. The opposite was observed for CCD4a #7 (Figure 2c). Although crocin production was higher in dark conditions in both lines seven days after subculture (Figure 2e), CCD2 #3 cells exhibited a slower growth rate under dark conditions, which was also observed in the wild‐type but not in CCD4a #7 (Figure 2f). Interestingly, the crocin profile was different between the lines and conditions assayed. The main crocins in CCD2 #3 under light and dark conditions were *trans*‐crocin‐2 and *trans*‐crocin‐2',respectively, whereas in CCD4a #7, the main crocin was always *trans*‐crocin‐3 (Table S2).

The CCD2 #3 line under light conditions was selected to perform further experiments, as it was the line that grew faster and did not exhibit significant differences in saffron apocarotenoid yield compared to CCD4a #7 under dark conditions. To determine the time point of the highest crocin yield, a growth curve was established under light conditions for the *N. benthamiana* CCD2 #3 cell suspension (Figure 2g; Figure S2). The highest crocin yield was observed in the late log phase, seven days after initiating the growth curve (Figure 2g).

### Generation of transgenic BY‐2 lines accumulating crocins, HTCC and picrocrocin

*N. tabacum* BY‐2 cells are the standard cell suspension system for plant research. Given the successful production of crocins in *N. benthamiana* cells, we aimed to utilize this established system to produce saffron apocarotenoids.

**Figure 3:**
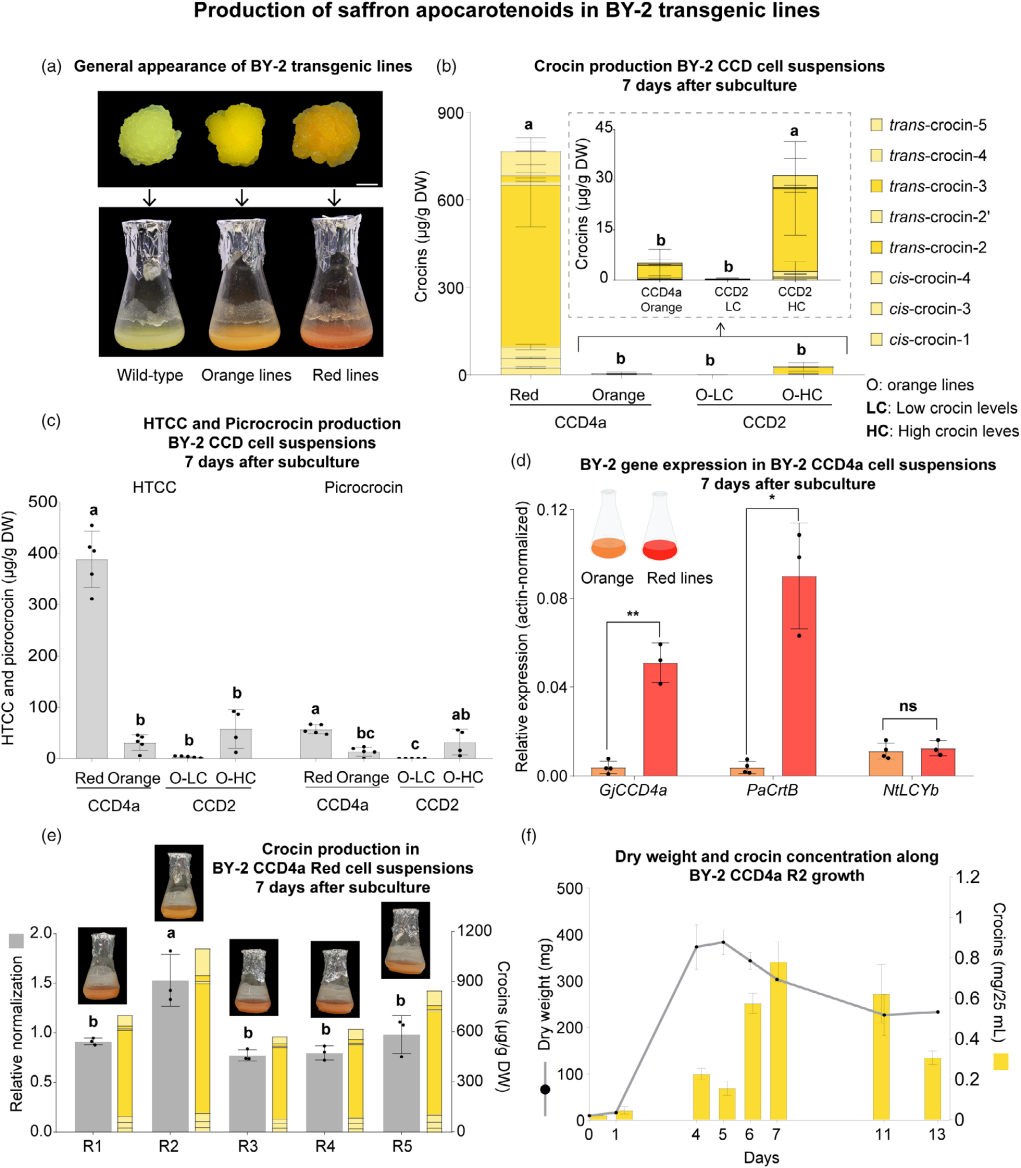
*Nicotiana tabacum* BY‐2 transgenic lines. (a) Phenotype of the calli and cell suspensions. Scale bar: 5 mm. (b) Crocin, (c) HTCC and picrocrocin production in BY‐2 7‐day‐old cell suspensions quantified by LC–MS. (d) Transgene expression in the BY‐2 CCD4a transgenic lines. (e) Relative and absolute crocin production in the different BY‐2 CCD4a red lines. (f) Dry weight and crocin concentration data from the growth curve of BY‐2 CCD4a R2. Further details of the growth curve can be found in Figure S3. Bars represent the mean ± standard deviation (SD), with individual points indicating biological replicates. Statistical differences between two conditions were determined using a Student's *t*‐test (non‐significant (ns), *P* ≤ 0.05 (*), *P* ≤ 0.01 (**) and *P* ≤ 0.001 (***)). For comparisons involving three or more samples, a one‐way ANOVA was performed, followed by Tukey's post hoc test. Different letters above the bars indicate statistically significant differences (*P* < 0.05) among groups. CCD: carotenoid cleavage dioxygenase. CrtB: bacterial phytoene synthase. LYCb: lycopene β‐cyclase. HTCC: 3‐OH‐β‐cyclocitral.

BY‐2 cells were transformed with the CCD2 and CCD4a constructs, and transgenic cell lines were selected visually based on the colour of the kanamycin‐resistant emerging calli, indicative of crocin or carotenoid accumulation. Forty‐five days after transformation, 40 CCD2 and 15 CCD4a calli showed yellow colour, while 37 CCD4a calli exhibited red colour. From these, ten CCD2 calli, five yellow CCD4a calli and five red CCD4a calli were selected and transferred to liquid medium. The yellow calli gave rise to orange cell suspensions, whereas the red calli resulted in red cell suspensions. The cell lines were named based on the colour of the suspensions (Figure 3a). All the transgenic lines exhibited growth characteristics similar to those of the wild‐type (Figure S3a).

Metabolite analyses revealed that all orange lines accumulated significantly lower levels of crocins, HTCC and picrocrocin than the red lines in DW (770 μg/g crocins, 390 μg/g HTCC, 57 μg/g picrocrocin) (Figure 3b,c). Among the CCD2 lines, some accumulated higher (31 μg/g DW), although still low, levels of crocins compared to the other lines (0.4 μg/g DW) (Figure 3b).

The differences among the CCD2 and CCD4a lines could be attributed to the expression levels of the transgenes. Higher crocin levels were associated with increased expression of *CsCCD2* among the CCD2 lines (Figure S3b), while the red CCD4a lines showed higher *GjCCD4a* expression compared to the orange CCD4a lines (Figure 3d). It appears that both low and high expression of *PaCrtB* result in carotenoid accumulation, but the CCDs require a minimum level of expression to produce high levels of saffron apocarotenoids in BY‐2 cells.

Among the CCD4a red lines, the CCD4a Red 2 (R2) cell line accumulated significantly higher levels of crocins than the other red cell lines (1.1 mg/g DW) (Figure 3e). CCD4a R2 was selected to establish a growth curve and determine the time point of maximum crocin yield. In BY‐2 CCD4a R2 cells, crocin concentration increased after reaching the maximum growth rate, 7 days after subculture (Figure 3f).

### Carotenoid accumulation in the *N. Benthamiana* and BY‐2 transgenic lines

The carotenoid profile of the transgenic lines from both platforms was evaluated to elucidate further the effect of exotic CCD expression on the accumulation of its substrates.

**Figure 4:**
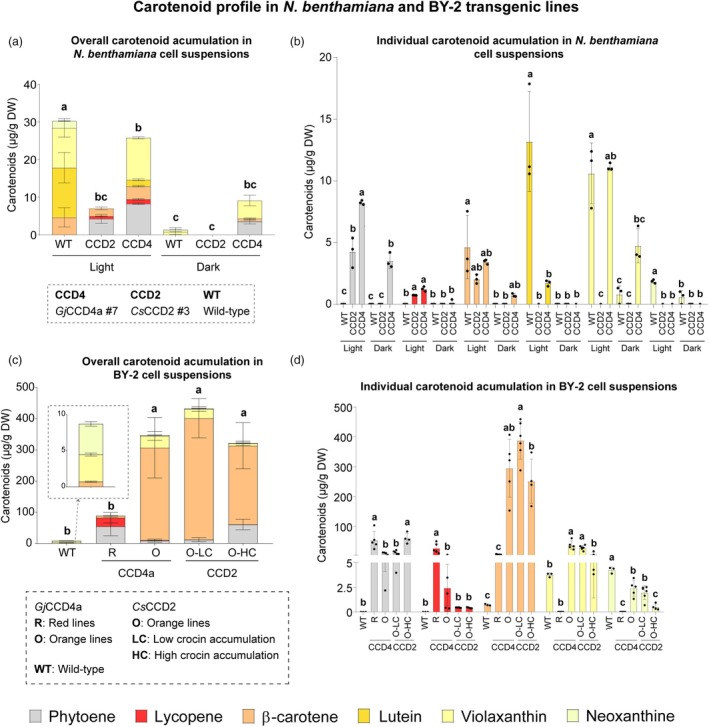
Carotenoid accumulation in 7‐day‐old wild‐type and CCD2 and CCD4a transgenic cell suspensions. (a) Total and (b) individual carotenoid accumulation in *N. benthamiana* BY‐2 cell suspensions. (c) Total and (d) individual carotenoid accumulation in *N. tabacum* cell suspensions. Carotenoids were quantified by HPLC‐PDA.

In *N. benthamiana*, the carotenoid profile was dramatically different from that of a typical leaf. Additionally, the carotenoid content of wild‐type cell suspensions displayed significant differences between light and dark conditions (Figure 4a). Under light conditions, carotenoid accumulation was high, with lutein and violaxanthin being the main carotenoids, while in darkness, only small amounts of violaxanthin and neoxanthin accumulated (Figure 4b). The transgenic lines showed dramatic differences compared to the wild‐type, primarily in the accumulation of phytoene in all cases except for CCD2 #3 under dark conditions, where no carotenoid accumulation was observed. However, the most striking finding was that under light conditions, both transgenic lines engineered with CCDs exhibited lycopene accumulation, which was not detected in any of the other cells (Figure 4a,b).

In light conditions, *N. benthamiana* CCD2 #3 did not accumulate lutein, violaxanthin or neoxanthin. In contrast, CCD4a #7 did not accumulate neoxanthin but lutein and violaxanthin, although the levels were lower than those in the wild‐type. In addition, this line also accumulated β‐carotene (Figure 4a,b).

The BY‐2 lines engineered with CCDs showed a dramatic carotenoid remodelling. The overall carotenoid content was significantly higher in the CCD lines than in the untransformed cells (Figure 4c) or all *N. benthamiana* cell suspensions (Figure 4a). Additionally, the differences in the carotenoid profile explained the different colours of the cell suspensions. CCD2 and CCD4a orange lines accumulated high levels of β‐carotene (295 μg/g DW) compared to the wild‐type (0.72 μg/g DW) and the CCD4a red lines (6.75 μg/g DW). The overall carotenoid accumulation in the CCD4a red lines was significantly lower than in all the other transgenic BY‐2 lines; they did not accumulate high levels of β‐carotene but instead accumulated lycopene and higher levels of phytoene than the CCD4a orange lines. CCD2 lines with higher crocin accumulation also displayed higher phytoene levels (Figure 4c,d).

### Production of saffron apocarotenoids in bioreactors

Following the successful production of saffron apocarotenoids in *N. benthamiana* and BY‐2 cells grown in flasks, we aimed to scale up the process. The *N. benthamiana* CCD2 #3 and BY‐2 CCD4a R2 lines were selected for cultivation in a wave bioreactor with a 10‐litre working volume.

**Figure 5:**
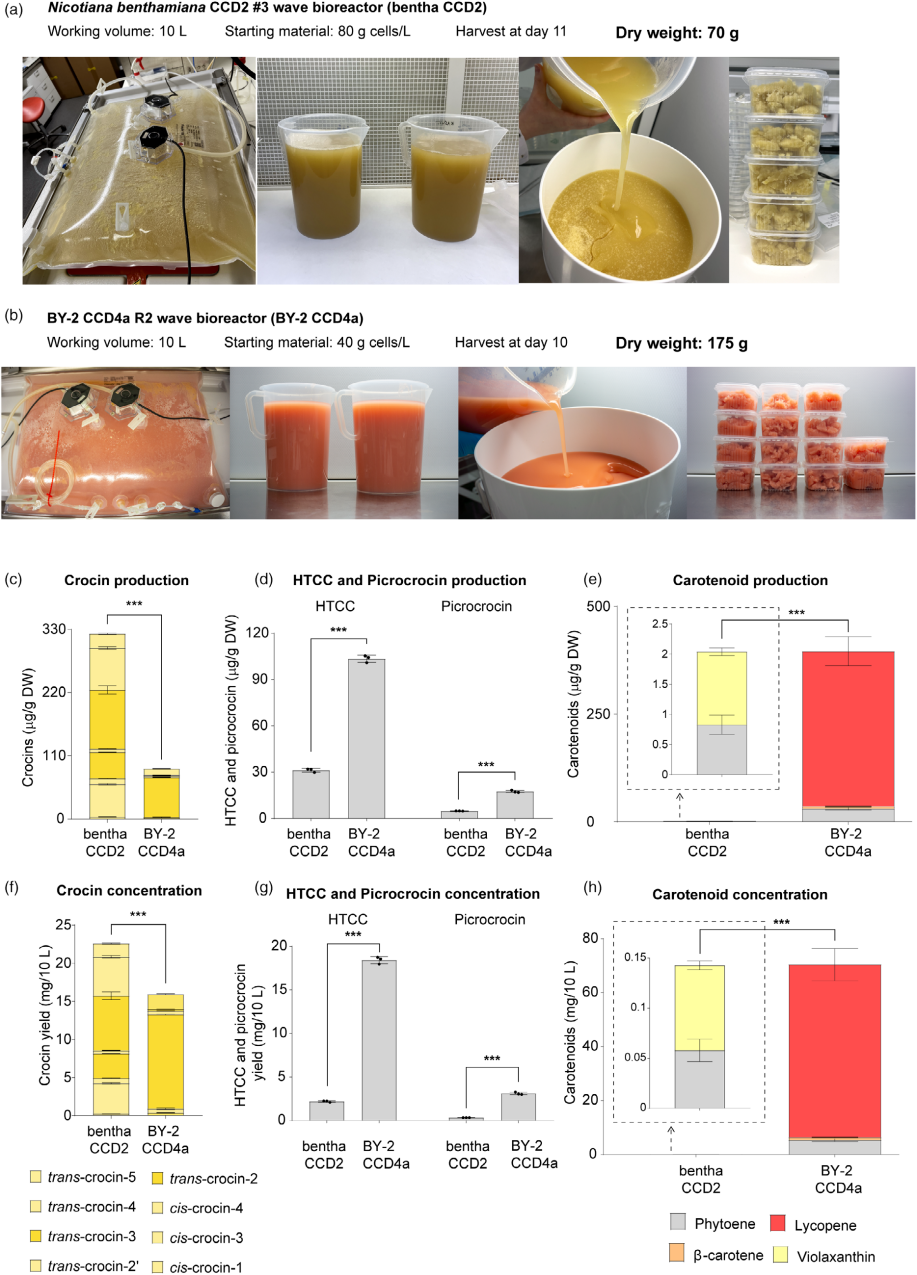
Growth of transgenic cell lines in wave bioreactors. (a) *N. benthamiana* CCD2 #3 bioreactor. (b) *N. tabacum* BY‐2 CCD4a R2 bioreactor. (c) Crocin, (d) HTCC, picrocrocin and carotenoid (e) production in the cells. (f) Crocin, (g) HTCC, picrocrocin and (h) carotenoid concentration in the 10 L working volume bioreactors. Crocins, HTCC and picrocrocin were measured by LC–MS. Carotenoids were measured by HPLC‐PDA. Statistical differences were determined using a Student's *t*‐test (*P* ≤ 0.001 (***)). HTCC: 3‐OH‐β‐cyclocitral.

The *N. benthamiana* CCD2 #3 bioreactor was harvested after 11 days, yielding 70 g of dry cells (Figure 5a). The BY‐2 CCD4a R2 wave bioreactor yielded 175 g of dry cells after 10 days (Figure 5b). Interestingly, the metabolite results obtained in the wave bioreactors differed from those obtained in flasks. For instance, while the saffron apocarotenoid production in *N. benthamiana* remained similar to that in flasks, the cells in the wave bioreactor accumulated violaxanthin instead of β‐carotene and lycopene. On the other hand, BY‐2 cells produced significantly less saffron apocarotenoids, especially crocins, and accumulated more lycopene than in the flasks (Figure 5c–e).

The crocin concentration was 25 and 16 mg of crocins from the 10 litres obtained from *N. benthamiana* and BY‐2 wave bioreactors, respectively (Figure 5f). Interestingly, the BY‐2 cells showed significantly higher concentrations of HTCC and picrocrocin than *N. benthamiana* cells (Figure 5g). In addition, BY‐2 showed a high concentration of carotenoids, mainly lycopene (64 mg), and *N. benthamiana* cells exhibited very low concentrations of carotenoids (0.15 mg) (Figure 5h).

### Elicitation of transgenic cell suspensions accumulating saffron apocarotenoids

After producing saffron apocarotenoids in both *N. benthamiana* and BY‐2 cells, we tested the effect of adding methyl jasmonate as an elicitor at the end of the exponential growth phase on crocin accumulation.

**Figure 6:**
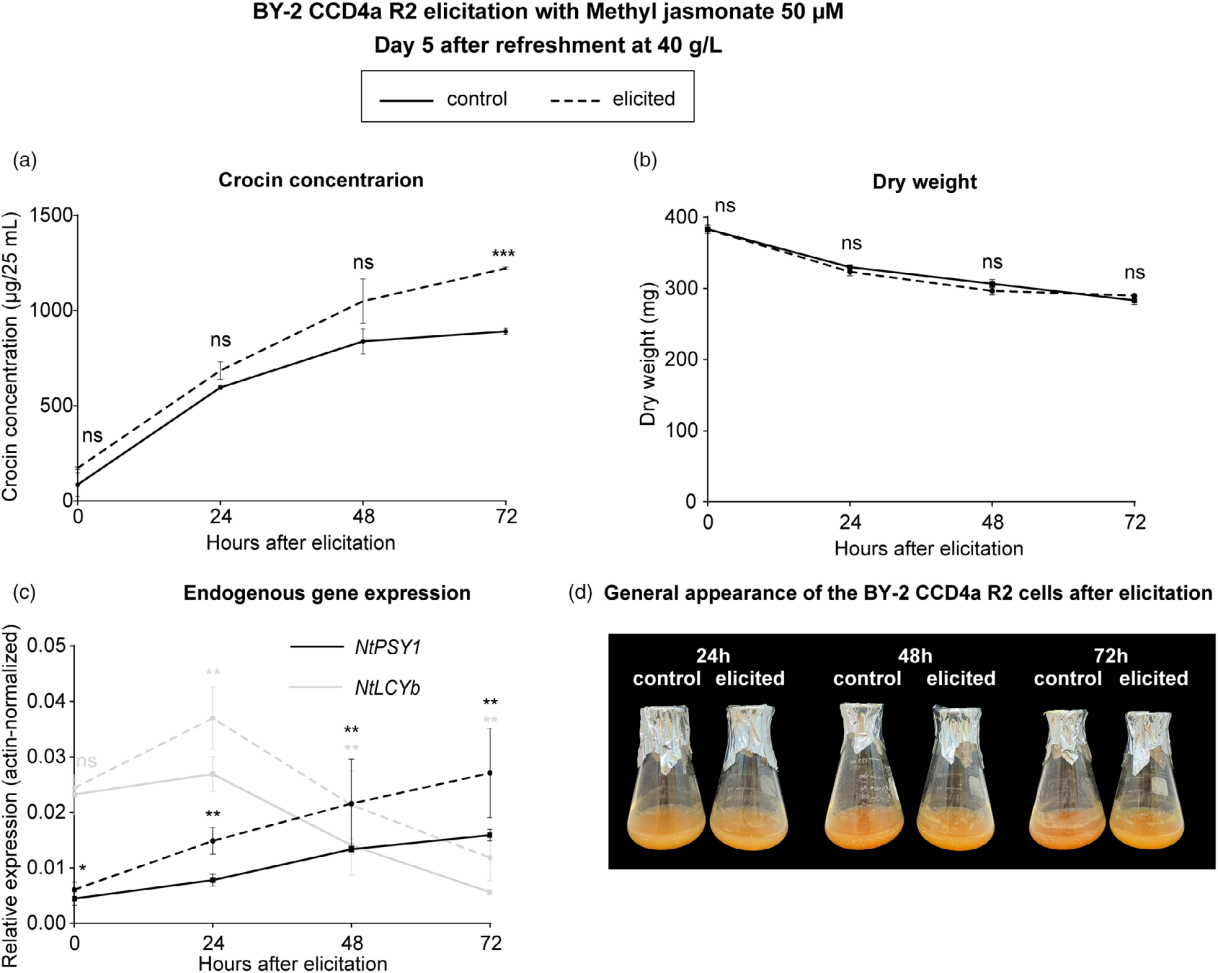
Elicitation of *N. tabacum* BY‐2 CCD4a R2 with methyl jasmonate. (a) Crocin concentration. (b) Dry weight. (c) Endogenous gene expression. (d) General appearance of the cells 24, 48 and 72 h after elicitation. Crocins were measured by Abs_443_. Each point represents the mean ± standard deviation (SD) from three biological replicates. Statistical differences between control and elicited conditions were determined using a Student's *t*‐test (non‐significant (ns), *P* ≤ 0.05 (*), *P* ≤ 0.01 (**) and *P* ≤ 0.001 (***)).

In BY‐2 CCD4a R2 cells, crocin concentration increased 1.4‐fold 72 h after elicitation, with no significant differences in crocin levels 48 h after elicitation (Figure 6a). This increase was attributed to higher crocin production and no effect on dry weight (Figure 6a,b). Gene expression of various endogenous carotenoid biosynthetic pathway genes was analysed at these two time points. The β‐carotene hydroxylase *NtCrtR‐1* was downregulated in the elicited samples compared to the control, while *NtPSY1* and *NtLCYb* were overexpressed (Figure S4b). These two genes also showed significant differences 24 h after elicitation, with *NtPSY1* even showing changes at 30 min post‐elicitation (Figure 6c). No significant changes in carotenoid accumulation were observed (Figure S4a).

Interestingly, in *N. benthamiana* cells, adding methyl jasmonate did not change crocin concentration or cell growth (Figure S4c,d).

### Changes in the volatile profile in BY‐2 cells accumulating saffron apocarotenoids

**Figure 7:**
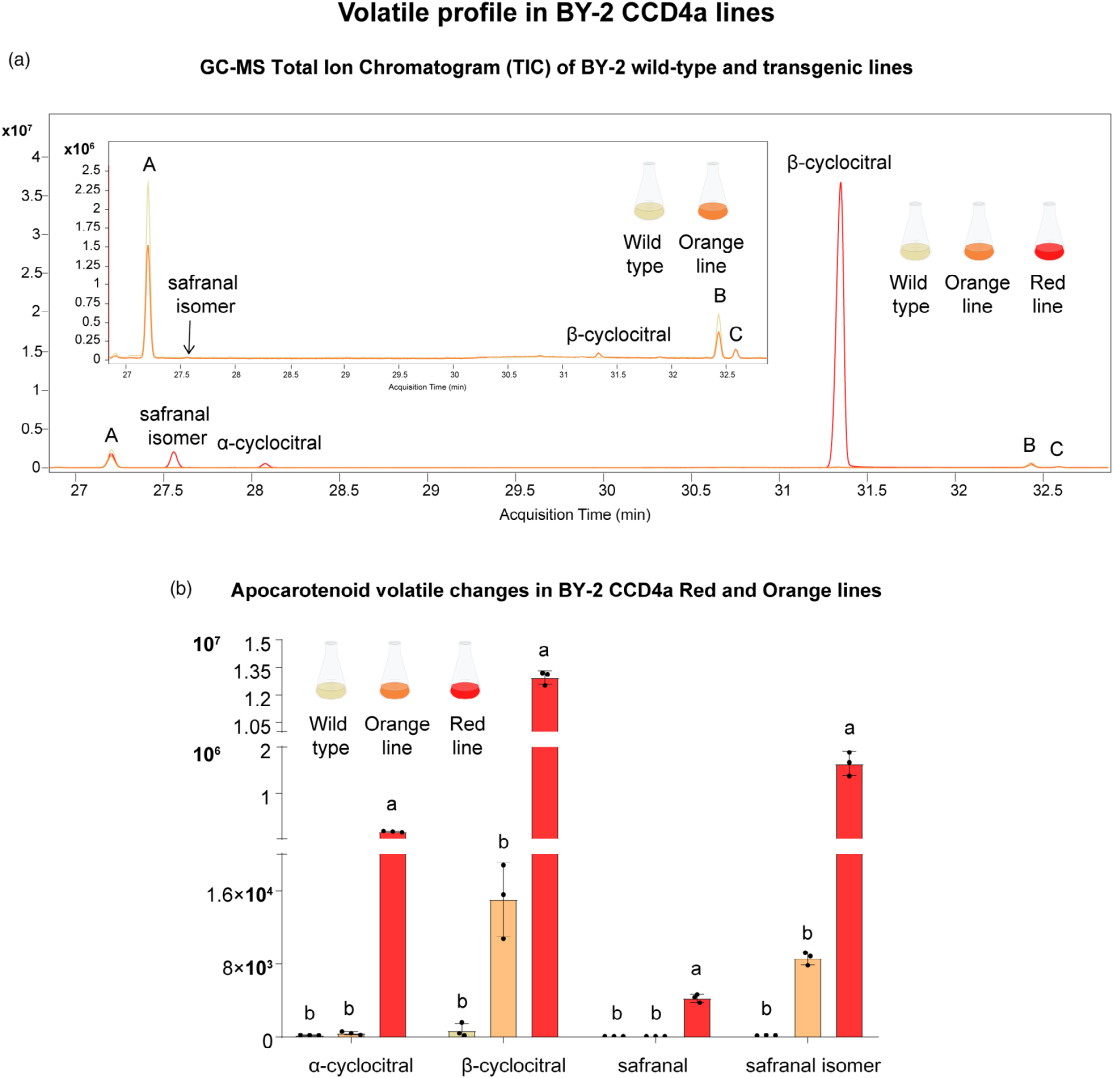
Volatile composition of BY‐2 wild‐type, CCD4a orange and red lines, determined by GC–MS. (a) Total Ion Chromatogram (minutes 27.0–32.5). The full‐time TIC chromatogram and the peak A, B and C identification can be found in Figure S5. (b) Volatile apocarotenoids. Bars represent the mean ± standard deviation (SD), with individual points indicating biological replicates. A one‐way ANOVA was performed, followed by Tukey's post hoc test. Different letters above the bars indicate statistically significant differences (*P* < 0.05) among groups.

The change in carotenoid composition and the expression of an exotic *CCD* in BY‐2 cells could lead to changes in volatile apocarotenoids derived from the cleavage of endogenous carotenoids. The volatile profiles of the red and orange BY‐2 CCD4a lines were compared with BY‐2 wild‐type cells (Figure 7). The volatile profile of the CCD4a red cells was dominated by volatile apocarotenoids. In particular, β‐cyclocitral was the main volatile compound produced in the red line, showing an 18 000‐fold increase (259.06 ± 7.34 μg/L) compared to the wild‐type, while in the orange line, it increased only 21‐fold (0.30 ± 0.08 μg/L) (Figure 7). The high amount of this volatile apocarotenoid was associated with the distinctive fruity smell detected in the saturated cells of the red CCD4a line, which was not perceived in other cell suspensions.

Changes were also observed in α‐cyclocitral, which increased by 900‐fold in the red lines and 2‐fold in the orange lines compared to the wild‐type. Neither the wild‐type nor the orange lines accumulated safranal, but the red line did. An unidentified volatile, likely a safranal isomer as it has the same molecular weight (MW = 150.21 g/mol), did not accumulate in wild‐type cells but was present in both CCD4a orange and red cells, with its amount in the red cells being 190 times higher than in the orange cells (Figure 7b).

## Discussion

### Saffron apocarotenoids accumulate to high levels in *N. Benthamiana* and BY‐2 cell cultures

In this study, two different plant cell culture platforms were developed to produce saffron apocarotenoids. In BY‐2 cells, *Gj*CCD4a outperformed *Cs*CCD2 when CCDs were expressed at high levels, resulting in 25, 7 and 2 times more crocins (770 vs. 31 μg/g), HTCC (408 vs. 58 μg/g) and picrocrocin (57 vs. 32 μg/g), respectively (Figure 3b,c). The red lines indicate that β‐carotene was the preferred substrate for *Gj*CCD4a, as β‐carotene was detected at very low levels (Figure 4c,d), and β‐cyclocitral was identified as the main volatile (Figure 7).

In contrast, in *N. benthamiana*, *Cs*CCD2 showed no significant differences compared to *Gj*CCD4a under light conditions, despite CCD4a #7 containing *PaCrtB* and CCD2 #3 lacking it (Figure 2c,e). Wild‐type *N. benthamiana* cell suspensions accumulate lutein under light conditions (Figure 4a,b), and it appears that both CCDs use this carotenoid as a substrate, as previously observed in transgenic tomato (Ahrazem *et al*., 2022a). However, lutein was not observed in tomato and CCD2 #3 cells, whereas it was present in CCD4a #7 cells (Figure 4a,b). Additionally, β‐carotene was still detected in transgenic CCD4a #7 suspensions (Figure 4a,b), similar to the findings in citrus callus expressing *GjCCD4a* (Zheng *et al*., 2022).

In flasks, crocin production in *N. benthamiana* CCD4a #7 was half that observed in BY‐2 CCD4a red lines (Figures 2e and 3b). Despite CCD4a #7 containing *PaCrtB*, the expression of this transgene is low compared to *GjCCD4a* (Figure S1e). The expression of *PaCrtB* in *N. benthamiana* transiently increased β‐carotene content efficiently (Llorente *et al*., 2020; Morelli *et al*., 2024) and has been used in combination with *CsCCD2* to enhance crocin accumulation in transient experiments (Martí *et al*., 2020). However, when introduced into plants under a constitutive promoter, some plants failed to integrate the transgenes, and those that did integrate them exhibited low expression levels (Figure S1b,d,e). The same constructs were used to generate transgenic BY‐2 lines, where all transgenes were successfully integrated into the plant DNA, and *PaCrtB* expression levels were similar to those of the *CCDs* (Figure 3d; Figure S3b).

Elicitation is another strategy to boost the carotenoid pathway and thereby enhance the production of saffron apocarotenoids. Methyl jasmonate enhanced carotenoid content in various platforms, including butterhead lettuce (Moreno‐Escamilla *et al*., 2020), broccoli (Nuñez‐Gómez *et al*., 2020), and cell suspension cultures of *Cleome rosea* (Silva da Rocha *et al*., 2015) and *Carum carvi* L. (Rahmati *et al*., 2023). This elicitor has also been used to enhance α‐tocopherol content in tomato and tobacco cell suspensions (Chandra *et al*., 2024; Harish *et al*., 2024). Additionally, salicylic acid has been shown to increase crocin accumulation in *C. sativus* cell suspensions by upregulating *β‐carotene hydroxylase* and *CCD2* expression (Moradi *et al*., 2022).

Even though the expression of *PaCrtB* already activated the carotenoid pathway, the BY‐2 transgenic cells elicited exhibited higher expression levels of *NtPSY* and *NtLCYb* compared to control samples and accumulated significantly greater amounts of crocins 72 h after elicitation (Figure 6). *LCYb* was also upregulated after elicitation in lettuce plants that produced more carotenoids (Moreno‐Escamilla *et al*., 2020). However, no changes were observed in the total carotenoid content of the BY‐2 CCD4a R2 cells (Figure S4a). In a previous study on BY‐2 elicitation with methyl jasmonate, a colour change toward orange was noted, suggesting an increase in β‐carotene content in elicited cells (Goossens *et al*., 2003). In *C. rosea*, β‐carotene content increased sixfold in elicited cells (Silva da Rocha *et al*., 2015). The elicitation with methyl jasmonate in our cells likely resulted in higher β‐carotene content, but *GjCCD4a* is consuming this carotenoid, thus explaining the increase in crocin production (Figure 6a).

Interestingly, elicitation did not lead to higher crocin accumulation in *N. benthamiana* CCD2 #3, where *PaCrtB* is absent (Figure S4d). Enhancing carotenoid and, thus, apocarotenoid content in *N. benthamiana* is more complex than expected, and alternative strategies should be explored to boost saffron apocarotenoid accumulation further.

### Remodelling carotenoid composition in BY‐2 transgenic cell cultures

The expression of *CsCCD2* in tomato fruits with different carotenoid contexts resulted in changes in the carotenoid profile, altering the composition of carotenoids that are not *Cs*CCD2 substrates (Lobato‐Gómez *et al*., 2024). The BY‐2 CCD4a red lines exhibited dramatic changes in carotenoid composition (Figure 4c,d). In this study, it was revealed that the yellowish colour of non‐transgenic BY‐2 cells is due to the low accumulation of violaxanthin and neoxanthin (Figure 4c,d); accumulation that is likely driven by the high levels of expression of the first enzyme involved in carotenoid hydroxylation, CrtR‐1 (Figure S4b). Unlike wild‐type BY‐2, which accumulates violaxanthin, or the other transgenic lines, which accumulate β‐carotene, the BY‐2 CCD4a red lines lacked both and accumulated significantly higher levels of lycopene. Interestingly, *N. benthamiana* transgenic cells in light conditions also accumulated low levels of lycopene (Figure 4a,b). Lycopene accumulation was also observed in tomato fruit expressing *CsCCD2* and carrying the *BETA* mutation, which prevents lycopene accumulation in fruits by its efficient conversion to β‐carotene (Lobato‐Gómez *et al*., 2024). The lycopene levels obtained in the BY‐2 bioreactor (360 μg/g DW) were not far from the 500 μg/g DW obtained in tomato calyx cell suspensions supplemented with CPTA, a lycopene β‐cyclase inhibitor (Robertson *et al*., 1995) and the yield (7 mg/L) was not far from the one obtained in *high‐pigment 1* tomato cell lines (up to 9 mg/L) (Moran *et al*., 2013). Interestingly, in *Barringtonia racemosa* cell suspensions, lycopene accumulation was significantly higher under light conditions, reaching concentrations up to 1.2 mg/g DW after five weeks of growth (Behbahani *et al*., 2011). The metabolic engineering of saffron apocarotenoid accumulation resulted in the production of lycopene, which could be further optimized.

Interestingly, BY‐2 CCD4a red lines and CCD2 lines with high *CsCCD2* expression, together with *N. benthamiana* transgenic cells (except CCD2 #3 in darkness), exhibited a significant increase in phytoene content (Figure 4). This increase in phytoene content was also observed in transgenic citrus calli expressing *CsCCD2* or *GjCCD4a* (Zheng *et al*., 2019), in *S. tuberosum* expressing *CsCCD2* (Gómez Gómez et al., 2022, 2024), *N. benthamiana* transiently expressing *CsCCD2* (Martí *et al*., 2020), and in tomato expressing *CsCCD2* in different carotenoid backgrounds (Lobato‐Gómez *et al*., 2024). This, together with the lycopene results, suggests that saffron apocarotenoids may regulate the carotenoid biosynthetic pathway independently of the platform and the CCD.

The orange BY‐2 lines also displayed an altered carotenoid profile compared to wild‐type cells. Both CCD4a and CCD2 lines with low CCD expression had similar carotenoid profiles, suggesting that the observed changes resulted from *PaCrtB* expression alone. These lines accumulated significantly higher β‐carotene and violaxanthin levels than the wild‐type (Figure 4c,d). Similar results have been reported in plant cell suspensions from citrus, *Iris germanica*, *Arabidopsis thaliana* and eggplant, where *PaCrtB* expression led to increased β‐carotene and xanthophyll levels compared to wild‐type (Cao *et al*., 2012; Jeknić *et al*., 2014; Maass *et al*., 2009; Mishiba *et al*., 2020). This suggests that phytoene synthase is the bottleneck for high levels of carotenoid accumulation, while the downstream phytoene carotenoid biosynthetic pathway is active in all these organisms.

Notably, *N. benthamiana* CCD2 #3 and BY‐2 CCD2 cells with high *CsCCD2* expression exhibited significantly lower violaxanthin levels than the wild‐type and the other orange lines, respectively (Figure 4b,d). This suggests that crocins are produced using zeaxanthin as a substrate, which would otherwise be converted to violaxanthin in cells not expressing exotic CCDs. In the red lines, violaxanthin was undetectable, indicating that *Gj*CCD4a consumes all available zeaxanthin before it can be converted to violaxanthin (Figure 4c,d).

Altogether, our results indicate that the expression of exotic CCDs can boost the production of high‐value carotenoids in well‐established cell suspension systems, even under non‐optimized conditions.

### Accumulation of volatile apocarotenoids in BY‐2 transgenic cells accumulating saffron apocarotenoids

The volatile profile of BY‐2 transgenic cells with low expression of *GjCCD4a* was similar to that of the wild‐type. Nevertheless, the cells from the BY‐2 CCD4a red line produced novel carotenoid‐derived volatiles, including safranal, a putative safranal isomer, α‐cyclocitral and extremely high amounts of β‐cyclocitral (Figure 7).

β‐cyclocitral, along with β‐ionone and dihydroactinidiolide, is generated through the oxidation of β‐carotene under stress conditions (Havaux, 2014; Ramel *et al*., 2012). β‐cyclocitral functions as a stress signal, modulating gene expression to shift plant cells from active growth to a defensive state. In *A. thaliana*, the oxidation of β‐carotene into β‐cyclocitral during simulated herbivore damage resulted in the suppression of the MEP pathway and the activation of defence signalling (Mitra *et al*., 2021). Similarly, in tomato plants, β‐cyclocitral application triggered metabolite changes that closely mirrored those induced by simulated herbivore damage (Deshpande and Mitra, 2023).

The elevated levels of β‐cyclocitral produced in the BY‐2 CCD4a red lines, likely from β‐carotene cleavage by *Gj*CCD4a, are particularly noteworthy as this volatile compound has demonstrated beneficial effects in plants when applied exogenously and therefore has value as an agrochemical. For instance, it has been shown to improve rice growth under salt‐contaminated soil conditions by regulating root architecture (Dickinson *et al*., 2019) and to enhance *A. thaliana* tolerance to photooxidative stress by influencing the expression of genes involved in oxidative stress response and cellular signalling (D'alessandro *et al*., 2018; Ramel *et al*., 2012).

The BY‐2 CCD4a red lines showed significantly fewer total carotenoids than the other BY‐2 transgenic lines (Figure 4c,d). In aquatic plants like duckweed, β‐cyclocitral at 0.05 to 2 mM concentrations suppresses growth and downregulates carotenoid biosynthesis (Du *et al*., 2022). However, in our transgenic lines, no growth penalties have been observed from the production of β‐cyclocitral (Figure S3a), and the low levels of carotenoids result from the high expression of *GjCCD4a*. In BY‐2 CCD2 lines, there is no decrease in carotenoid accumulation despite the high expression of *CsCCD2* (Figure 4c) because β‐carotene is not used as a substrate for this enzyme (Frusciante *et al*., 2014). In tomato, an introgression line with higher expression levels of *CCD4b* exhibited an increase in phytoene and a decrease in lycopene and β‐carotene, which serve as substrates for CCD to produce geranylacetone and β‐ionone (Yoo *et al*., 2023), showing similar behaviour to BY‐2 CCD4a red lines, where lycopene accumulates because it is not used as a substrate for *Gj*CCD4a and, interestingly, it is not converted to β‐carotene by LCYb.

Taken together, BY‐2 CCD4a red cells constitute a plant‐based alternative for producing β‐cyclocitral, which is usually synthesized chemically in a contained and sustainable manner.

### Upscaling the production of saffron apocarotenoids in plant cell suspensions

The results of this work represent a first step toward upscaling saffron apocarotenoid production in heterologous plant cell suspension systems to an industrial scale. In *C. sativus* cells, the maximum crocin yield achieved in flasks was 0.80 mg/g DW when the cells were harvested at the end of the sixth week after subculturing. The concentration of crocins obtained in the flasks was 2.74 mg/L of cell suspension (Amini *et al*., 2022). In our system, *N. benthamiana* CCD2 #3 and BY‐2 CCD4a red lines produced 0.30 mg/g and 0.75 mg/g DW of crocins, respectively, after one week of growth, with dry weight increasing threefold in *N. benthamiana* and 40‐fold in BY‐2 within five days (Figures 2g and 3f). Although crocin production per dry weight of cells is lower in our system, the maximum crocin concentration achieved in flasks was 3.4 mg/L and 14.2 mg/L in *N. benthamiana* CCD4a #7 (dark) and BY‐2 CCD4a R2 (Figures 2c,g and 3b,f), respectively. Additionally, these yields were achieved seven days after subculturing. Overall, the production in our system is faster and results in higher crocin yields than in *C. sativus* cell suspensions.

Heterologous proteins have been produced in *N. benthamiana* (Huang *et al*., 2010; Huang and McDonald, 2009) and BY‐2 (Raven *et al*., 2015; Schmale *et al*., 2006) cultivated in bioreactors. We evaluated the performance of our transgenic lines in a 10‐L wave bioreactor (Figure 5) and successfully produced crocins, HTCC and picrocrocin in this system. Due to the different growth conditions in flasks and bioreactors, the metabolite levels and profile were not the same on both platforms. The lower saffron apocarotenoid production in wave bioreactors suggests that plant cells need extra time to adapt to the new growth conditions and produce saffron apocarotenoids.

Crocin concentration was much lower in BY‐2 than in *N. benthamiana* (Figure 5f), likely due to differences in crocin accumulation patterns during cell growth in both platforms. In *N. benthamiana*, crocin accumulation increases as cells grow (Figure 2g), while in BY‐2, crocin production begins once cell growth slows down (Figure 3f). Since cell monitoring relied on dry weight data compared to growth curves prepared in flasks, considering that the growth rate may differ between flask and wave bioreactor, there is a high probability that the bioreactors were not harvested at the optimal time. For *N. benthamiana*, crocins are produced throughout the entire growth phase, so harvesting a day earlier or later than the ideal point would not lead to substantial differences. In contrast, harvesting the BY‐2 bioreactor too early may mean the cells were still growing, resulting in lower crocin accumulation.

Although the yields were not optimal, our results indicate that producing saffron apocarotenoids in larger volumes is possible. Optimization of the *N. benthamiana* CCD lines and BY‐2 CCD4a R2 growth should be carried out to further upscale the production of saffron apocarotenoids. Many growth parameters, including pH, dissolved oxygen and cell density, can be controlled in stirred‐tank bioreactors but not in wave bioreactors (Huang and McDonald, 2012). Despite wave bioreactors allowing the upscaling from small volumes and preventing cross‐contamination by the use of disposable bags (Lehmann *et al*., 2014; Wierzchowski and Pilarek, 2024; Zhou *et al*., 2010), the next step to optimize the production of saffron apocarotenoids in cell suspensions should be to monitor their growth in stirred‐tank bioreactors. Most conventional stirred‐tank systems, especially at larger scales, do not allow light conditions. Therefore, *N. benthamiana* CCD4a #7 should be used instead of CCD2 #3 due to its higher yields in dark conditions.

Transgenic BY‐2 and *N. benthamiana* cell suspension cultures were successfully developed as heterologous platforms for producing saffron apocarotenoids. BY‐2 CCD4a red lines additionally accumulated lycopene and high levels of β‐cyclocitral, a volatile apocarotenoid with agronomic relevance, without growth penalties. Meanwhile, *N. benthamiana* CCD lines efficiently produced crocins, picrocrocin and HTCC in dark and light conditions while accumulating low levels of lycopene when exposed to light. These systems exhibited fast growth rates, making them promising alternatives for biotechnological production. Furthermore, successful cultivation in a 10‐L wave bioreactor demonstrated the scalability of these platforms, with stirred‐tank bioreactors identified as promising candidates for further optimization. These findings lay the groundwork for the large‐scale production of valuable apocarotenoids and carotenoids in plant cell cultures.

## Experimental procedures

### Plant material

*N. benthamiana* LAB strain was used. Wild‐type and transgenic plants were grown in a greenhouse at 21 °C with a 16/8 h photoperiod. *N. benthamiana* cell suspensions were grown under two different conditions: at 24 °C in dark conditions and 26 °C in light conditions (16/8 h photoperiod), always at 130 rpm.

*N. tabacum* cv. BY‐2 cell suspension cultures were used to perform cell suspension transformation. BY‐2 wild‐type and transgenic cells were grown at 28 °C, darkness and 150 rpm.

### Construct assembly

The constructs were assembled following the GoldenBraid (GB) strategy (Vazquez‐Vilar *et al*., 2021). The transcriptional units for *C. sativus CCD2*, *G. jasminoides CCD4a*, *Pantoea ananatis CrtB* and *C. sativus UGT91P3*, under the control of the 35S promoter, were assembled in the GB destination vectors. Then, two final modules were obtained by combining the different TUs: *P35S*:*CsCCD2*_*P35S*:*PaCrtB*_*P35S:CsUGT91P3*_*nptII* (CCD2) and *P35S*:*GjCCD4a*_*P35S*:*PaCrtB*_*P35S:CsUGT91P3*_*nptII* (CCD4a) (Figure S1a). The cloning steps were performed in *Escherichia coli*, and the final CCD2 and CCD4a constructs were electroporated into *A. tumefaciens* LBA4404.

### Plant transformation

*N. benthamiana* leaf explants were transformed with the CCD2 and CCD4a constructs. Young leaves were collected and surface‐sterilized with 70% ethanol for 10 s and 5% sodium hypochlorite for 10 min. Then, leaf explants were placed on MS media supplemented with 1 mg/L 6‐benzylaminopurine (BAP) and 0.1 mg/L naphthalene acetic acid (NAA) for 24 h. The explants were infected with *A. tumefaciens* (OD_600_ 0.1) and co‐cultivated with *A. tumefaciens* for 48 h. Then, the explants were placed on selection media (MS supplemented with BAP, NAA, 100 mg/L kanamycin and 200 mg/L carbenicillin) and transferred weekly until shoot formation. The shoots that developed roots on MS supplemented with kanamycin were genotyped for transgene presence using the primers listed on Table S1 and transferred to the greenhouse.

BY‐2 cells were transformed with both CCD2 and CCD4a constructs. BY‐2 cells were diluted 1:10 seven days after subculture and grown at 28 °C for 3 days. The *A. tumefaciens* preculture was prepared 2 days prior to the transformation. Then, 3 mL of BY‐2 cells were placed on Petri dishes, and 200 μL of *A. tumefaciens* preculture were added. After 3 days of inoculation, the cells were plated on selection media (MS media supplemented with 0.2 mg/L 2,4‐D, 0.1 g/L myo‐inositol, 1 mg/L thiamin‐HCl, 0.2 g/L KH_2_PO_4_, 50 mg/L kanamycin, 100 mg/L vancomycin and 250 mg/L carbenicillin). After 3 weeks, the potential transgenic calli were transferred to fresh selection media.

### Establishment of transgenic plant cell suspensions

Leaf explants from the *N. benthamiana* transgenic plants, confirmed for crocin accumulation in leaves, were used to induce callus formation. The explants were surface‐sterilized as described above, placed on callus induction media (solid MS supplemented with 0.5 mg/L 2,4‐D and 0.2 mg/L kinetin) and transferred biweekly to fresh medium. Once the callus was generated and exhibited suitable friability, the cell suspensions were started by adding four 1‐centimetre diameter calli to 20 mL of liquid callus induction media. Homogeneous cell suspensions were obtained after several weekly subcultures by diluting them 1:2 with fresh media.

In BY‐2 transformation, calli displaying new colour hues due to transgene expression were selected and transferred to fresh media biweekly. The BY‐2 transgenic cell suspensions were established by adding two 1‐centimetre diameter calli into 20 mL of BY‐2 liquid selection media. Cell suspensions were obtained after weekly subcultures by diluting them 1:5 with fresh media. After five transfers to selection media, vancomycin and carbenicillin were removed. After 10 transfers into selection media, kanamycin was removed.

### Measurement of callus and cell suspension growth

To measure callus growth, three plates per genotype and/or condition were prepared, each containing callus pieces of approximately 0.25 cm diameter. After two weeks, four random calli from each plate were collected to measure fresh weight.

To assess cell suspension growth, a known volume of the cultures was taken from the flasks, vacuum‐filtered and collected to determine fresh weight. Dry weight was measured after freeze‐drying the material.

For *N. benthamiana*, a growth curve was established at 26 °C under light conditions, starting with 80 g/L of fresh cells. For BY‐2, the growth curve was established at 28 °C, starting with 40 g/L of fresh cells. The cells were distributed into flasks with 25 mL of cell suspension.

For each time point of the growth curve, three biological replicates were analysed. Fresh weight and dry weight were measured. The pH was measured at each time point, as well as the sugar content, which was analysed using a Dionex ICS‐6000 HPLC–MS (Thermo Fisher, Massachusetts, USA). Total crocin content was assessed at each time point by measuring absorbance at 443 nm, and the crocin yield was calculated by multiplying the absorbance values by the dry weight.

### Elicitation

The elicitation experiments were conducted using methyl jasmonate (Duchefa Biochemie, Haarlem, The Netherlands) at a final concentration of 50 μM per flask. In both platforms, elicitation followed the same conditions as the growth curve preparation. It was initiated at the late exponential phase based on growth curve data (day 5 in *N. tabacum* BY‐2 and day 7 in *N. benthamiana*), and samples were collected 30 min, 24, 48 and 72 h post‐elicitation. Control samples without elicitation were also collected. For each time point, three biological replicates were analysed.

### Upscaling

A wave bioreactor (Biostat RM, Sartorius, Germany) was initiated from each platform with the best cell suspension line based on crocin yield and the dry weight 7 days after subculture. In both cases, the working volume was 10 litres, using 20‐litre disposable plastic bags (CultiBag RM, 20 L basic, Sartorius, Germany).

For *N. benthamiana*, the bioreactor was started with a cell concentration of 80 g/L. The wave bioreactor was operated under light conditions (RX400, Valoya, Finland) with a 16/8 h photoperiod at 26 °C and a rocking speed of 24 rpm.

For BY‐2, the starting concentration was 40 g/L of cells. The wave bag was maintained in darkness at 28 °C, with a rocking speed of 24 rpm.

Harvesting time was determined based on fresh weight measurements and visual inspection. Cell suspensions were vacuum‐filtered, weighed and frozen at −20 °C. Total dry weight was calculated after lyophilization.

### Metabolite analyses

Saffron apocarotenoids and carotenoids were extracted from 10 mg of freeze‐dried material following an established protocol (Perez‐Fons *et al*., 2022). The polar fraction was used to determine total crocin accumulation by measuring absorbance at 443 nm, subtracting the values of non‐transgenic samples. Measurements were performed using a Varioskan Lux microplate reader (ThermoFisher, USA). The polar fraction was also used to quantify crocin, 3‐OH‐β‐cyclocitral (HTCC) and picrocrocin by LC–MS, using genistein as an internal standard and crocin‐4 standard curve (Ahrazem *et al*., 2022a), while the non‐polar fraction was analysed for carotenoids by HPLC‐PDA, using canthaxanthin as an internal standard (Lobato‐Gómez *et al*., 2024). The yield of the metabolites was calculated by considering the total dry weight.

In the case of BY‐2 cells, volatile organic compounds (VOCs) were captured from 1 mL of saturated cell suspensions (7 days after subculture, at 80 g/L), following a previously described protocol (Rambla and Granell, 2020) with slight modifications. Samples were taken directly from the flasks and transferred to screw‐cap headspace vials to evaluate the VOCs produced during cell growth. The samples in the vials were pre‐incubated for 10 min at 30 °C with orbital shaking, and the VOCs were adsorbed to a 65 μm PDMS/DVB SPME fibre for 20 min under the same conditions. Then, the volatiles were desorbed and injected into a gas chromatograph for analysis. The average value of all samples was used to normalize the data for relative quantification. A standard curve with the specific volatile compound was used for absolute quantification.

### Gene expression analyses

RNA was extracted from the freeze‐dried leaf, callus and cell suspension material using the GeneJET Plant RNA Purification Kit (ThermoFisher, USA), followed by DNA digestion with the TURBO DNA‐free™ Kit (ThermoFisher, USA). cDNA synthesis was performed using the PrimeScript 1st Strand cDNA Synthesis Kit (Takara, Shiga, Japan). PCR reactions were carried out with the TB Green Premix Ex Taq II (Takara, Japan) on a QuantStudio 3 system (ThermoFisher, USA). Primers used for gene expression analyses are listed in Table S1.

### Statistical analysis

All statistical analyses were conducted using R software. The choice of statistical tests for assessing significant differences between samples or groups of samples was based on data distribution, the number of outliers and variance differences between groups. The specific tests used are detailed in the figure legends.

## Conflicts of interest

The authors declare no conflicts of interest.

## Author contributions

A.G., H.R. and D.O. designed the research. M.L.G, M.L., M.V.V. and J.L.R. performed the experiments. M.L.G. analysed the data and wrote the article. All authors approved the final version.

## Supporting information


**Table S1** GoldenBraid pieces used and generated, and primers used in this study.
**Table S2** Crocin percentage in *Nicotiana benthamiana* CCD lines.
**Figure S1** Constructs, PCR and RT‐qPCR results of *Nicotiana benthamiana* CCD lines.
**Figure S2** Data collection of *Nicotiana benthamiana* CCD2 #3 growth curve.
**Figure S3** Data collection of BY‐2 CCD lines, including the data collection of BY‐2 CCD4a R2 growth curve.
**Figure S4** Data collection from the elicitation of the CCD cell suspensions with methyl jasmonate 50 μM.
**Figure S5** Volatile composition BY‐2 lines determined by GC–MS.

## Data Availability

The data supporting the findings of this study are available within the manuscript and its Supporting Information.
