# Supplementary material for: Novel plant cell suspension platforms for saffron apocarotenoid production and its impact on carotenoid and volatile profiles
Source: Plant Biotechnol J. 2025 Jun 19;23(9):3903–18. doi: 10.1111/pbi.70153 (PMC12392949; doi:10.1111/pbi.70153)
Supplement: Supplementary file 1 — Table S1 GoldenBraid pieces used and generated, and primers used in this study. Table S2 Crocin percentage in Nicotiana benthamiana CCD lines. Figure S1 Constructs, PCR and RT‐qPCR results of Nicotiana benthamiana CCD lines. Figure S2 Data collection of Nicotiana benthamiana CCD2 #3 growth curve. Figure S3 Data collection of BY‐2 CCD lines, including the data collection of BY‐2 CCD4a R2 growth curve. Figure S4 Data collection from the elicitation of the CCD cell suspensions with methyl jasmonate 50 μM. Figure S5 Volatile composition BY‐2 lines determined by GC–MS. [file PBI-23-3903-s001.docx]

**Supporting Information**

Novel plant cell suspension platforms for saffron apocarotenoid production and its impact on carotenoid and volatile profiles

Table S1. GoldenBraid pieces used and generated, and primers used to genotype the putative transgenic plants and to perform gene expression analyses. *Cs*: *Crocus sativus*; *Gj*: *Gardenia jasminoides*; *Pa*: *Pantoea ananatis*; *Nt*: *Nicotiana tabacum*. CrtB: bacterial phytoene synthase. UGT: UDP-glycosyltransferase. CCD: carotenoid cleavage dioxygenase. PSY: phytoene synthase. LCYb: lycopene β-cyclase. Crt-R: β-carotene hydroxylase. nptII: neomycin phosphotransferase II.

| **GoldenBraid plasmids** | | |
| --- | --- | --- |
| GB_number | Name | Type of plasmid |
| GB30 | pUPD_P35S | PROMOTER |
| GB36 | pUPD_T35S | TERMINATOR |
| GB226 | pEGB1alpha1R_TNOS:*nptII*:PNOS | TRANSCRIPTIONAL UNIT |
| GB4658 | pUPD2_*PaCrtB* | CDS |
| GB4659 | pUPD2_*CsUGT91P3* | CDS |
| GB4858 | pUPD2_*CsCCD2L* | CDS |
| GB4794 | pUPD2_*GjCCD4a* | CDS |
| GB5011 | pDGB3a1_P35S:*GjCCD4a*:T35S | TRANSCRIPTIONAL UNIT |
| GB5012 | pDGB3a2_P35S:*PaCrtB*:T35S | TRANSCRIPTIONAL UNIT |
| GB5013 | pDGB3a2_P35S:*CsUGT91P3*:T35S | TRANSCRIPTIONAL UNIT |
| GB5050 | pDGB3o1_P35S:*GjCCD4a*_P35S:*PaCrtB*_P35S: *CsUGT91P3*_*nptII* | MODULE |
| GB5051 | pDGB3o1_P35S:*CsCCD2L*_P35S:*PaCrtB*_P35S: *CsUGT91P3*_*nptII* | MODULE |
|  |  |  |
|  |  |  |
| **Primers for transgene amplification** | | |
| Gene | FW | RV |
| *CsCCD2* | CATTTGGAGAGGACTCCGGT | GCAAACTTGGGGTTAGGACC |
| *GjCCD4a* | CATTTGGAGAGGACTCCGGT | TGATTGCGGCTTGGGATTGG |
| *PaCrtB* | CATTTGGAGAGGACTCCGGT | GTAAGCCGGGGCGATATCAT |
| *CsUGT91P3* | CATTTGGAGAGGACTCCGGT | GAGGTACTGGAGGTTTGCCG |
|  |  |  |
| **Primers for gene expression analyses** | | |
| Gene | FW | RV |
| *NtPSY1* | AATTAGCACAGGCAGGGCTT | TCTTGCGATACAACAGCAGC |
| *NtPSY2* | TGGCCTGTATGGGCATCTTT | GGGGCACAAGAGATTTTGCAT |
| *NtLCYb* | AAGGAGACGTCAACGAGAGT | GAGAAAAGGGACAGCCCGAAA |
| *NtCrtR-1* | GGTTCACAAGAGATTCCCCGT | CCCCCTACTTCTTCCAATTCCTT |
| *NtCCD4* | GGATGAGGACGATGGCTACG | AAAGGCCGTGGAAACCGTAA |
| *Ntactin* | CGGAATCCACGAGACTACATAC | GGGAAGCCAAGATAGAGC |
| *CsCCD2* | ACATGTCGCCTTGAGAGTCC | TCAGATTTGATGCCAGGTTG |
| *GjCCD4a* | ATCCCAAGCCGCAATCATCA | TTCCACCACCTCACATTCGG |
| *PaCrtB* | GTCGAAACGATGGCAGTTGG | CGTTGTTCGGGCGTTTGTAA |
| *CsUGT91P3* | TAGGCCTACTGCTGCCTACT | GCAGGATCTCGGTATCGCTC |

Table S2. Crocin percentage in *Nicotiana benthamiana* CCD lines. A one-way ANOVA was performed, followed by Tukey’s post hoc test. Different letters indicate statistically significant differences (p < 0.05) among groups. CCD: carotenoid cleavage dioxygenase.

|  | CCD2 #3 Light | | | CCD2 #3 Dark | | | CCD4a #7 Light | | | CCD4a #7 Dark | | |
| --- | --- | --- | --- | --- | --- | --- | --- | --- | --- | --- | --- | --- |
|  | Mean | SD |  | Mean | SD |  | Mean | SD |  | Mean | SD |  |
| *cis*-crocin-1 | 2.3 | 0.15 | b | 16.7 | 2.13 | a | 1.27 | 0.27 | b | 0.38 | 0.02 | b |
| *trans*-crocin-2 | **27.24** | 1.58 | a | 11.65 | 6.57 | b | 18.51 | 0.94 | b | 17.16 | 0.5 | b |
| *trans*-crocin-2' | 2.41 | 0.2 | b | **41.56** | 1.87 | a | 1.52 | 0.05 | b | 2.01 | 0.35 | b |
| Low-glycosylation | 31.95 |  |  | 69.91 |  |  | 21.3 |  |  | 19.55 |  |  |
| *cis*-crocin-3 | 19.68 | 0.28 | a | 6.37 | 1.81 | b | 17.77 | 0.12 | a | 4.53 | 0.4 | b |
| *trans*-crocin-3 | 24.64 | 0.38 | c | 9.88 | 2.05 | d | **40.77** | 0.7 | b | **46.27** | 0.23 | a |
| *cis*-crocin-4 | 3.29 | 0.17 | a | 3.28 | 0.04 | a | 2.02 | 0.1 | b | 1.2 | 0.02 | c |
| *trans*-crocin-4 | 13.77 | 0.56 | b | 9.81 | 0.27 | c | 14.42 | 0.58 | b | 21.14 | 0.24 | a |
| *trans*-crocin-5 | 6.68 | 0.67 | a | 0.75 | 1.05 | c | 3.72 | 0.16 | b | 7.31 | 0.18 | a |
| High-glycosylation | 68.06 |  |  | 30.09 |  |  | 78.7 |  |  | 80.45 |  |  |


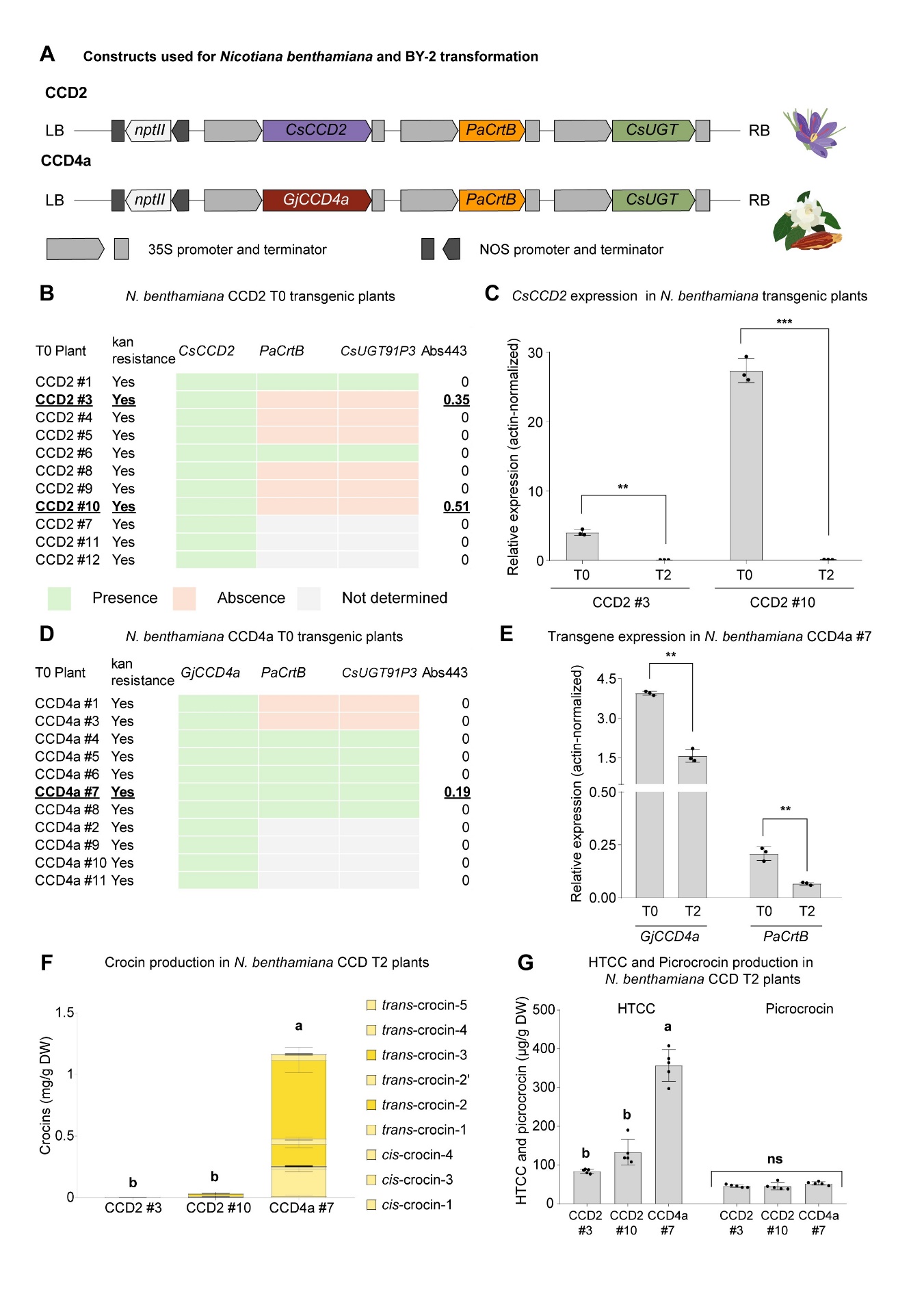


Figure S1. **A**: Constructs used to transform *Nicotiana benthamiana* leaf explants and BY-2 cells. *Cs*: *Crocus sativus*; *Pa*: *Pantoea ananatis*; *CsUGT*: *CsUGT91P3*; nptII: neomycin phosphotransferase II enzyme; LB: left border; RB: right border. **B**: Genotyping results in *N. benthamiana* CCD2 lines and absorbance at 443 nm to measure crocin accumulation. **C**: *CsCCD2* expression results in transgenic T0 and T2 *N. benthamiana* plants from lines CCD2 #3 and #10. **D**: Genotyping results of *N. benthamiana* CCD4a lines and absorbance at 443 nm. **E**: Transgene expression results in transgenic T0 and T2 *N. benthamiana* CCD4a #7 plants. **F**: Crocin production in *N. benthamiana* T2 plant leaves. **G**: HTCC and picrocrocin accumulation in *N. benthamiana* T2 plant leaves. Crocin, HTCC, and picrocrocin were quantified by LC-MS. Bars represent the mean ± standard deviation (SD), with individual points indicating biological replicates. Statistical differences between conditions or samples were determined using a Student’s t-test. Significance levels are indicated as non-significant (ns), p ≤ 0.05 (*), p ≤ 0.01 (**), and p ≤ 0.001 (***). For comparisons involving three or more samples, a one-way ANOVA was performed, followed by Tukey’s post hoc test. Different letters above the bars indicate statistically significant differences (p < 0.05) among groups. nptII: neomycin phosphotransferase II. HTCC: 3-OH-β-cyclocitral. CCD: carotenoid cleavage dioxygenase, CrtB: bacterial phytoene synthase. UGT: UDP-glycosyltransferase.


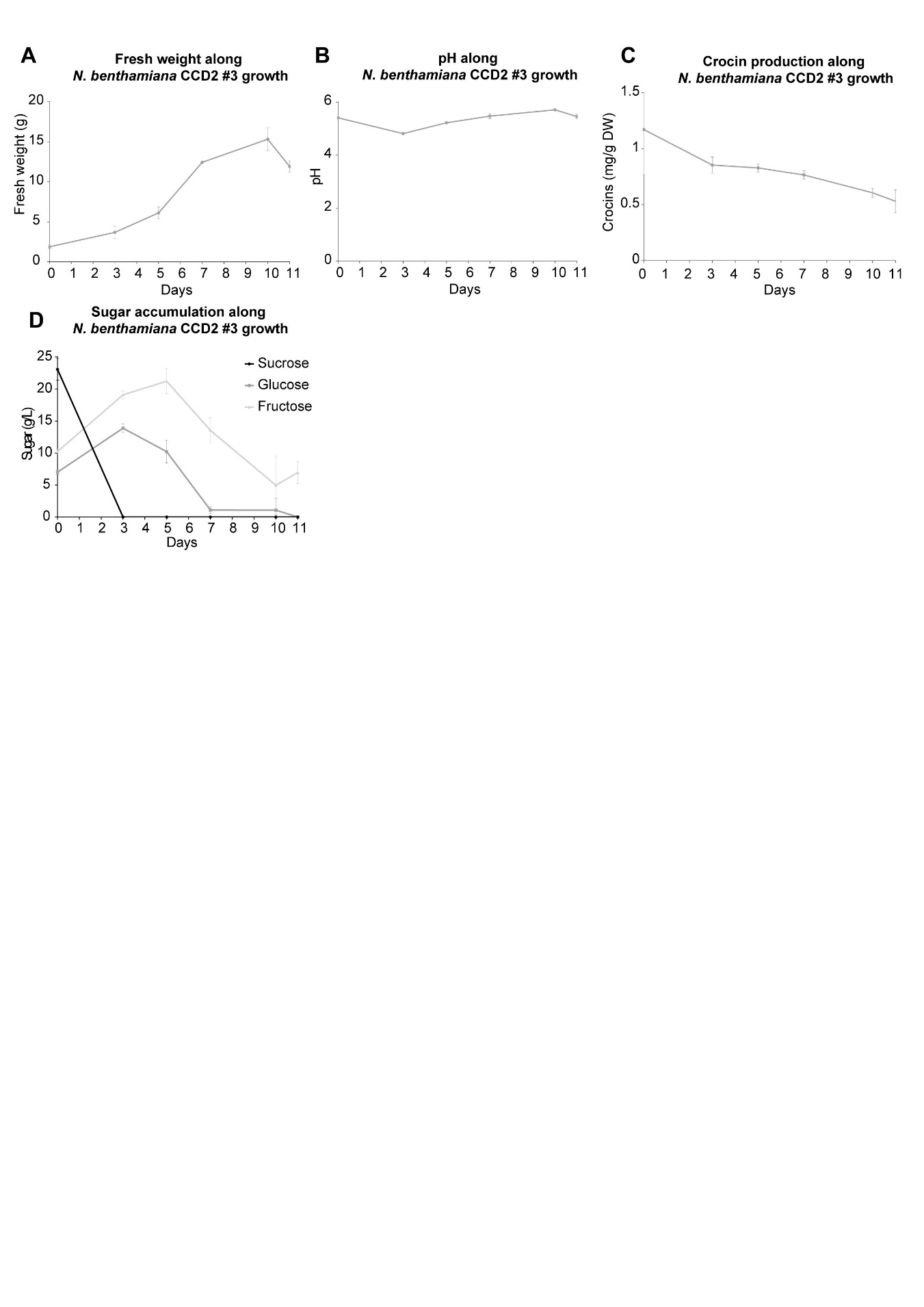


Figure S2. *Nicotiana benthamiana* CCD2 #3 growth curve data collection. **A**: Fresh weight. **B**: pH along the growth curve. **C**: Crocin production quantified by measuring Abs_443_. **D**: Sugar content measured by HPLC-MS. Each point represents the mean ± standard deviation (SD). CCD: carotenoid cleavage dioxygenase.


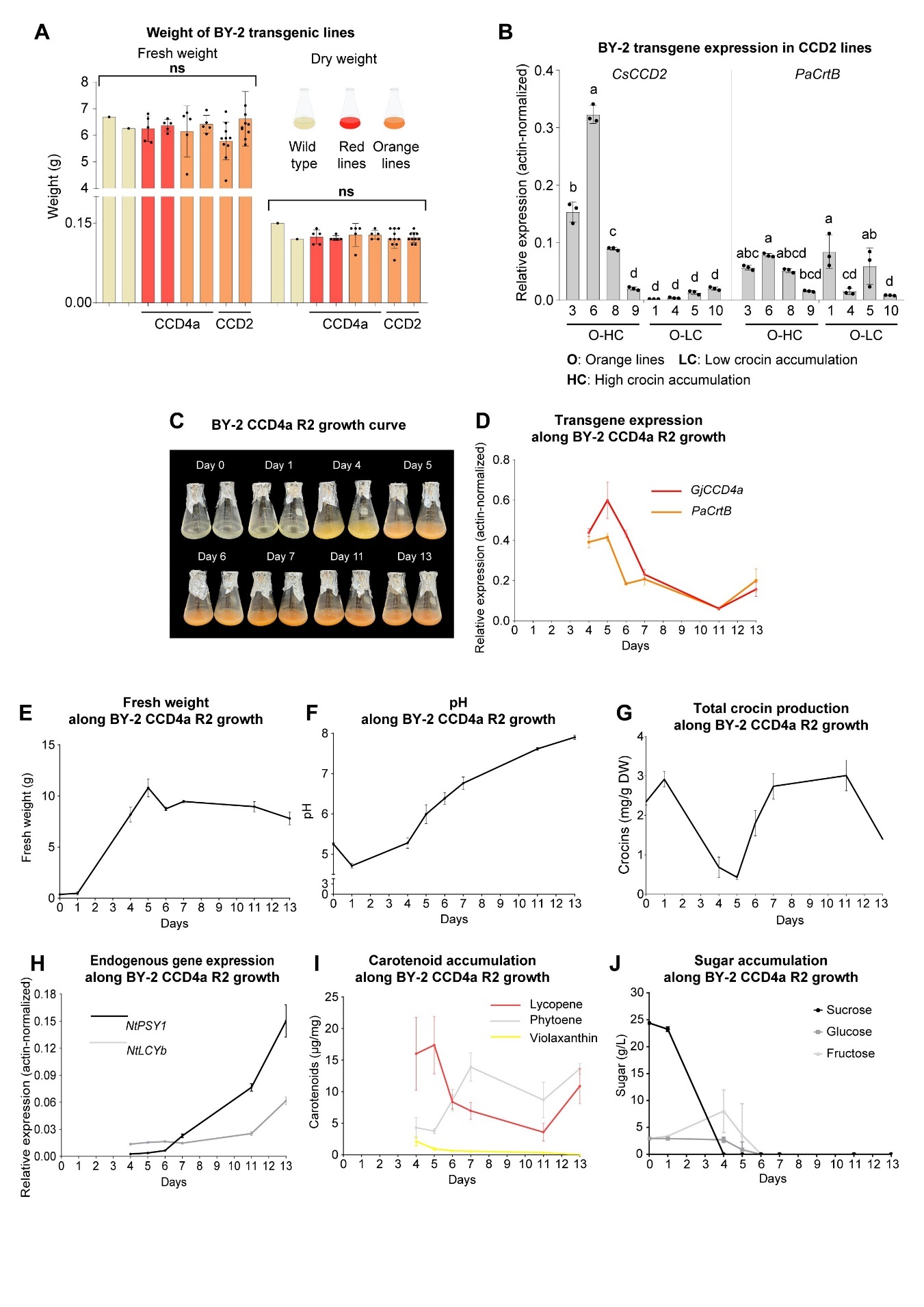


Figure S3. BY-2 cell suspensions **A**: Fresh and dry weight of BY-2 wild type and transgenic lines. **B**: Transgene expression in BY-2 CCD2 lines. BY-2 CCD4a R2 growth curve parameters assessed at different time points: (**C**) general appearance, (**D**) transgene expression, (**E**) fresh weight, (**F)** pH, (**G**) total crocin production, quantified by measuring Abs_443_, (**H**) endogenous gene expression, (**I**) carotenoid accumulation, measured by HPLC-PDA), and (**J**) sugar content measured by HPLC-MS. Bars represent the mean ± standard deviation (SD), with individual points indicating biological replicates. A one-way ANOVA was performed, followed by Tukey’s post hoc test. Different letters above the bars indicate statistically significant differences (p < 0.05) among groups. CCD: carotenoid cleavage dioxygenase. CrtB: bacterial phytoene synthase. PSY: phytoene synthase. LCYb: lycopene β-cyclase.


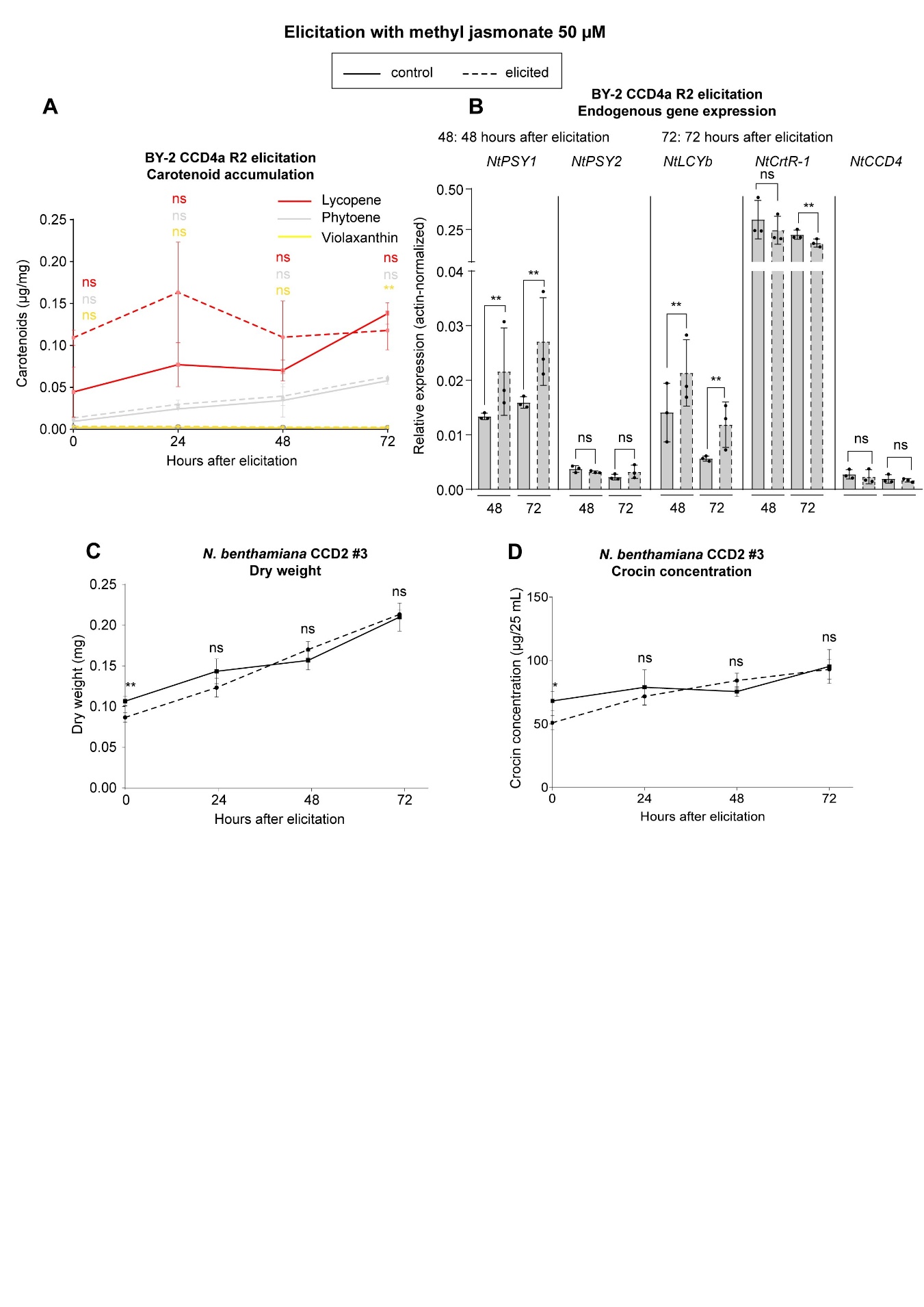


Figure S4. Elicitation with methyl jasmonate 50 µM. (**A**) Carotenoid accumulation was quantified by HPLC-PDA, and (B) endogenous gene expression in *Nicotiana tabacum* BY-2 CCD4a R2 (elicitation started on day 5 after subculture). (**C**) Dry weight, (**D**) crocin concentration in *Nicotiana benthamiana* CCD2 #3 (elicitation started on day 7 after subculture). Crocins were measured by Abs_443,_ subtracting the value of the wild type. Each point and bar represents the mean ± standard deviation (SD) from three biological replicates. Statistical differences between control and elicited conditions were determined using a Student’s t-test (non-significant (ns), p ≤ 0.05 (*), p ≤ 0.01 (**), and p ≤ 0.001 (***)). PSY: phytoene synthase. LCYb: lycopene β-cyclase. Crt-R: β-carotene hydroxylase. CCD: carotenoid cleavage dioxygenase.


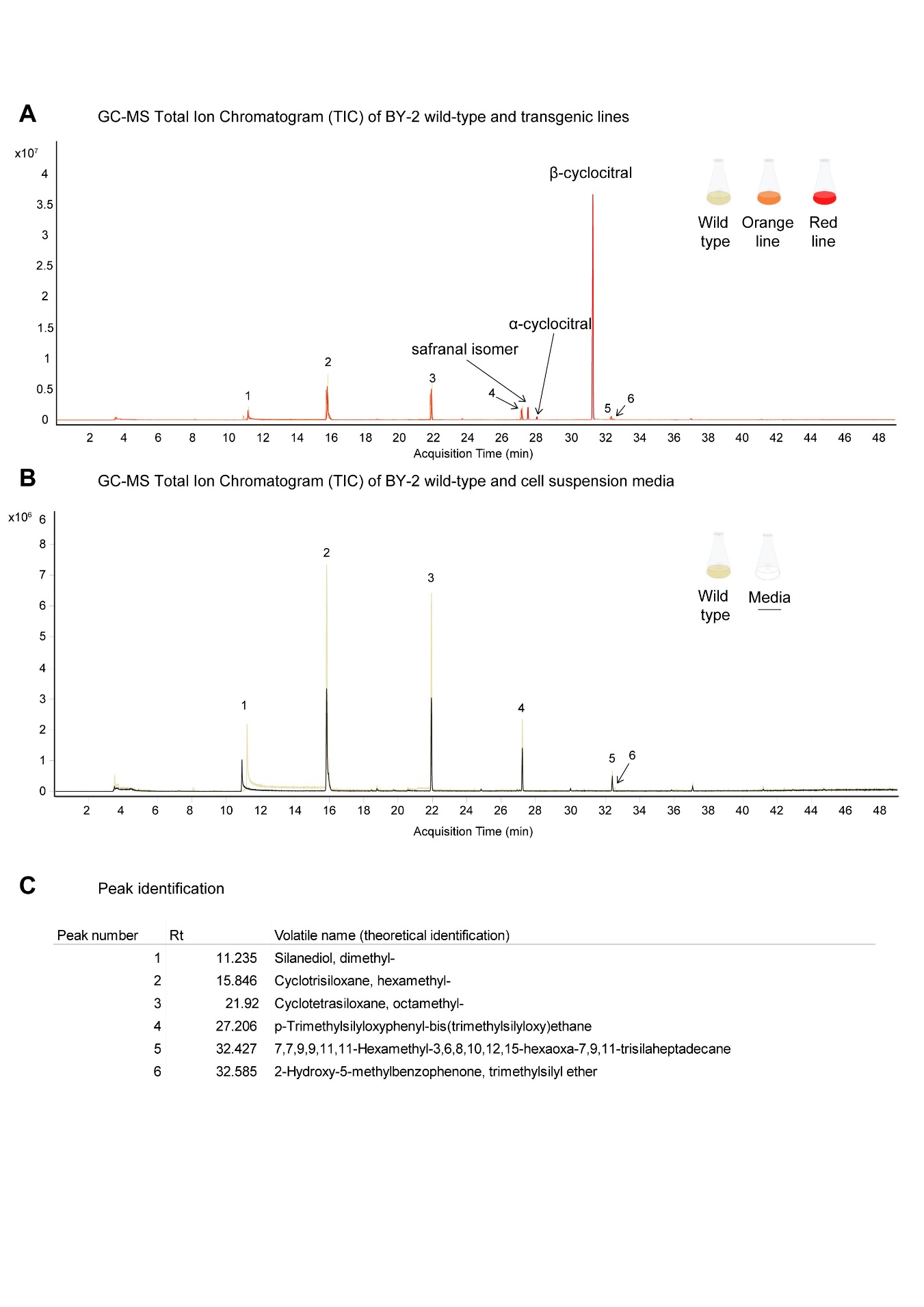


Figure S5. Volatile composition *Nicotiana tabacum* BY-2 lines determined by GC-MS. **A:** Total Ion Chromatogram (TIC) BY-2 wild-type, BY2-2 CCD4a orange (O3), and BY-2 CCD4a red (R2). **B**: TIC BY-2 wild-type and BY-2 media. **C**: Tentative peak identification according to the National Institute of Standards and Technology (NIST).
